# Editorial: School Achievement and Failure in Portuguese and Spanish Speaking Countries

**DOI:** 10.3389/fpsyg.2018.01074

**Published:** 2018-06-25

**Authors:** Edgar Galindo, Adelinda A. Candeias, Heldemerina S. Pires, Miguel Ángelv Carbonero

**Affiliations:** ^1^Center for Research in Education and Psychology (CIEP), Psychology, Universidade de Évora, Évora, Portugal; ^2^Excellence Research Group GR179, Educational Psychology, University of Valladolid, Valladolid, Spain

**Keywords:** school achievement, school failure, academic achievement, educational psychology, applied psychology

School failure is an important social problem in the present world (Organization for Economic Co-operation Development, 2010). This seems to be especially true for Spanish and Portuguese speaking countries in Europe (Eurydice, 2011) and Latin America (Román, 2013). Consequently, school achievement and failure is an increasingly important topic of discussion and research. Psychology can make an important contribution to understanding these issues, emphasizing the individual perspective, i.e., defining academic and pre-academic skills for a good achievement and developing sound evaluation and intervention techniques for children with school failure problems. The following papers revise the latest developments in the field. The research covered tried to answer the most urgent questions on the topic: (1) Which variables are responsible for school achievement and failure? (2) Which intervention techniques can be used to help children with school failure problems?

All papers focused on children attending primary or secondary school, except one that dealt with prevention strategies for children at risk before schooling. Most sought correlations between determining variables and some measures of school achievement. Two studies applied intervention strategies designed to improve the academic performance of children with problems at school and systematically evaluated their results.

Oriol et al. observed the effect of self-control and grit on academic self-efficacy and satisfaction with school, using structural equation models. Both variables showed strong associations in both primary and secondary students. Grit is related to academic-self efficacy at both educational stages, but it is only correlated with satisfaction with school in secondary students. Self-control was only significantly related to school satisfaction in primary education. Oliveira et al. explored the links between career preparedness and academic development and how both varied across demographic characteristics during middle school years, employing gender and geographical location as potential moderators affecting the link between the domains of career and school. Results showed a longitudinal relationship between the school and work in youth.

Three studies focused on emotional variables. Franco et al. observed the relationship between emotion understanding and school achievement, considering age, gender, fluid intelligence, mother's educational level and social competence. A structural equation model revealed that the relationship between emotion understanding and school performance depended on a mediator variable designated as social competence. Mothers' educational level predicted social emotional competence and fluid intelligence was a predictor of emotion understanding, school achievement, and social emotional competence. A similar analysis was conducted by López et al. using structural equation modeling to observe the association of individual and school-related well-being variables with school achievement and performance in children who answered questionnaires assessing subjective wellbeing, social wellbeing at school, school climate, school social wellbeing and students' perceptions of teachers' wellbeing. Results showed that students' individual subjective wellbeing was associated with their achievement and performance in school. Cristóvão et al. reviewed the implementation in Portugal of programs to foster social and emotional learning (SEL), which was defined as the capacity to recognize and manage emotions, solve problems effectively, and establish positive relationships with others, and their results on academic achievement. After consulting several databases, they concluded that little such research is being produced in Portugal and what is carried out it scattered across many journals and authors.

Three more studies investigated the variables influencing academic success in specific subject matters. Marcelino et al. explored the role of early numerical competencies (ENC) (counting, number relations, and basic arithmetic operations) in early mathematics learning. Using a regression model, this study analyzed the relation between ENC and mathematics achievement in first-grade children. Results showed that children with low numerical competencies performed less well than those with moderate and high numerical competencies. Findings suggested that ENC are meaningful for predicting first-grade mathematics difficulties. Vilia et al. analyzed the impact of two variables on academic achievement in Physics-Chemistry, namely, student's attitudes toward the subject and reasoning abilities (measured respectively through the Attitude toward Physics-Chemistry Questionnaire/ATPCQ, and the Reasoning Test Battery/ RTB). An assessment using multiple regression stepwise analyses and standardized regression coefficients revealed ATPCQ and RTB to be significantly related to students' achievement in Physics and Chemistry. Pires et al. explored parental support as a variable determining success in a Portuguese language course. As expected, results showed a clear influence of parental support on academic achievement, but significant differences appeared according to gender, with girls being more sensitive to the affective dimension of parental support and boys to the instrumental one.

In his study of prevention measures applied in Portugal to children at risk, Franco et al. explained how the National System of Early Intervention contributed to promoting maximum development and the full inclusion of children up to 6 years of age, thus reducing the likelihood of school failure.

Last but not least, two studies applied psychology when implementing scientifically based intervention techniques for children with academic problems. Carbonero et al. carried out an intervention program based on Don Hellison's Positive Action Theory and Fishbein and Ajzen's Theory of the Reasoned Action, focusing on (a) teaching units on social responsibility, (b) student training in mediation processes, (c) teacher training, and (d) family training. Results showed that students schooled in social responsibility performed significantly better than those in a control group. Social responsibility seems to be related to commitment, self-discipline, and perseverance. Galindo et al. applied Behavioral Skills Training (BST), consisting of instructions, modeling, rehearsal, and feedback, to teach basic behavior (precurrents), academic behavior, or social behavior to a group of children with school failure problems, in two different studies. Results from the first study, using pre-test/post-test measures, showed an increase in academic achievement. A second study, using a multiple baseline design, showed few performance changes without training and academic improvement following BST. In both studies, comparable results occurred across students, demonstrating the positive effects of the intervention.

The approaches discussed in this Research Topic provide insight for teachers and psychologists so that they might better understand school achievement and failure, while it also serves as a source of potential tools for intervention in cases of school failure.

## Author contributions

All authors listed made a substantial, direct and intellectual contribution to the work and approved it for publication.

### Conflict of interest statement

The authors declare that the research was conducted in the absence of any commercial or financial relationships that could be construed as a potential conflict of interest.
